# Resilience Buddy: A Multi-System Approach to Mental Well-Being

**DOI:** 10.1192/j.eurpsy.2026.10743

**Published:** 2026-07-31

**Authors:** K. P. Fung, S. Kim

**Affiliations:** 1 University of Toronto, Toronto; 2 McMaster University, Hamilton, Canada

## Abstract

**Introduction:**

There is increasing recognition of the need to broaden the scope of psychiatry to include primary prevention. Emerging frameworks integrate well-being science, social determinants of health, lifestyle psychiatry, and the cultivation of cultural and structural competence to address issues of equity, diversity, and inclusion (EDI). Within this context, resilience is increasingly understood as a key protective factor that can enhance mental well-being, reduce vulnerability to psychiatric illness, and promote recovery.

**Objectives:**

This project aims to translate the Multi-System Model of Resilience (MSMR) into an accessible, evidence-informed digital platform that empowers individuals to strengthen resilience across multiple domains.

**Methods:**

The MSMR conceptualizes resilience as a dynamic, relational process shaped by biological, psychological, social, cultural, and ecological systems. It emphasizes that resilience is not simply an inherent trait but the outcome of interactions across multiple layers of influence. Within this framework, MSMR describes three interrelated systems: (1) **Internal Resilience**, grounded in physiological regulation and psychological skills; (2) **Values and Coping**, which bridges internal and external systems through meaning-making, problem-solving, and flexibility; and (3) **External Resilience**, which encompasses family, community, structural, and ecological resources. Drawing from this model, *Resilience Buddy* was designed as an app comprising nine structured modules. Each module integrates interactive psychoeducational activities, experiential exercises, and brief guided meditations tailored to build capacities across the three resilience systems.

**Results:**

A preliminary pilot with five participants yielded encouraging results, with positive feedback regarding both usability and acceptability, measured using the Usability Metric for User Experience (UMUX). Participants highlighted the clarity, accessibility, and relevance of the content. Recruitment is currently underway for a larger pilot trial with 30 participants. This study will provide additional data on feasibility, user experience, and preliminary outcomes related to resilience enhancement. Detailed findings, including module design, engagement metrics, and preliminary outcome data, will be presented at the conference.

**Conclusions:**

*Resilience Buddy* represents an innovative, scalable approach to promoting mental well-being through a multi-system resilience framework. By situating resilience within interacting biological, psychological, social, and structural contexts, it challenges individualizing or pathologizing narratives and promotes culturally sensitive, equity-oriented strategies for prevention. Preliminary findings suggest that digital, interactive resilience training is both feasible and engaging, with the potential to complement existing mental health interventions and strengthen population-level mental health promotion.

**Disclosure of Interest:**

None Declared

